# Buprenorphine Alters Inflammatory and Oxidative Stress Molecular Markers in Arthritis

**DOI:** 10.1155/2017/2515408

**Published:** 2017-05-09

**Authors:** Mahadevappa Hemshekhar, Vidyanand Anaparti, Carol Hitchon, Neeloffer Mookherjee

**Affiliations:** ^1^Manitoba Centre for Proteomics and Systems Biology, University of Manitoba, Winnipeg, MB, Canada; ^2^Department of Internal Medicine, University of Manitoba, Winnipeg, MB, Canada; ^3^Department of Immunology, University of Manitoba, Winnipeg, MB, Canada

## Abstract

Buprenorphine is recommended for use as an analgesic in animal models including in murine models of collagen-induced arthritis (CIA). However, the effect of buprenorphine on the expression of disease-associated biomarkers is not well defined. We examined the effect of buprenorphine administration on disease progression and the expression of inflammatory and oxidative stress markers, in a murine model of CIA. Buprenorphine administration altered the expression of cytokines, IFN-*γ*, IL-6, and MMP-3, and oxidative markers, for example, iNOS, superoxide dismutase (SOD1), and catalase (CAT), in the CIA mice. As buprenorphine is an analgesic, we further monitored the association of expression of these biomarkers with pain scores in a human cohort of early rheumatoid arthritis (RA). Serum MMP-3 levels and blood mRNA expression of antioxidants *sod1* and *cat* correlated with pain scores in the RA cohort. We have demonstrated that administration of buprenorphine alters the expression of inflammatory and oxidative stress-related molecular markers in a murine model of CIA. This caveat needs to be considered in animal experiments using buprenorphine as an analgesic, as it can be a confounding factor in murine studies used for prediction of response to therapy. Furthermore, the antioxidant enzymes that showed an association with pain scores in the human cohort may be explored as biomarkers for pain in future studies.

## 1. Introduction

Buprenorphine is a potent opioid derivative of the alkaloid thebaine that binds to opiate receptors and prevents the sensation of pain. Therefore, it is used in the treatment of acute and chronic pain [1]. Buprenorphine is used in veterinary medicine to manage pain associated with tissue inflammation, necrosis and spasms and in ischemia and trauma resulting from wounds, fractures, and joint injuries [2]. Consequently, buprenorphine is recommended by institutional ethics boards for experimental animal models of various diseases to prevent and/or manage pain [3]. Easing of pain in laboratory mice is an essential requirement of in vivo research, particularly in models of inflammatory diseases such as rheumatoid arthritis (RA). Despite the recommended use of buprenorphine to manage pain in animal experimentation, there are no studies to date that detail the effects of buprenorphine administration on molecular markers of inflammation in murine models used for preclinical studies. Therefore, it is essential to delineate whether buprenorphine used as an analgesic interferes with the pathogenesis of the disease and/or alters the expression of disease-associated biomarkers in an animal model. Previous studies have shown that molecular mechanisms of anti-inflammatory and analgesic effects may be linked in animal models of inflammatory diseases such as arthritis [4, 5]. This highlights the importance of defining the effect of analgesics such as buprenorphine in an animal model, as administration of buprenorphine may be a confounding factor in preclinical studies focused on determining the underlying molecular mechanisms of candidate therapeutics.

In this study, we used a well-characterized murine model of collagen-induced arthritis (CIA) as previously described [6], to examine the effect of buprenorphine administration on disease progression and the expression of disease-associated serum and synovial biomarkers. The CIA model is the most extensively used preclinical animal model of RA, as it elicits autoimmunity to collagen type II (CII), inflammation of synovial joints, cartilage destruction, and bone erosion [7, 8]. Several studies have demonstrated the anti-inflammatory effects of buprenorphine on rat models of various inflammatory diseases, including those in an adjuvant-induced arthritis model [9]. In this study, we showed that administration of buprenorphine as an analgesic altered the expression of specific molecular markers of inflammation and those related to oxidative stress response in a murine model of CIA.

Administration of buprenorphine altered synovial mRNA expression of certain inflammatory mediators, matrix metalloproteinase *mmp-3*, oxidative stress marker, *iNos*, and specific antioxidants, and altered serum concentration of specific cytokines in the murine model of CIA. As buprenorphine is an analgesic, we further assessed the association of expression of the molecular markers that were altered with buprenorphine administration in the murine model of CIA, with pain scores in a human cohort of early RA. We showed that serum levels of inflammatory mediators such as MMP-3 and blood mRNA expression of antioxidant enzymes superoxide dismutase (*sod1*) and catalase (*cat*) significantly correlated with pain scores in the human early RA cohort. Overall, in this study, we define the effects of buprenorphine administration on the expression of disease-associated molecular markers in a murine model of CIA. Our results suggest that the use of buprenorphine as an analgesic in animal models may be a confounding factor for assessment of underlying molecular mechanisms, such as those in the processes of inflammation and oxidative stress. In addition, our observational results in the human cohort open new avenues of research to explore the use of the molecular indicators defined in this study as potential biomarkers for pain in future studies.

## 2. Materials and Methods

### 2.1. Collagen-Induced Arthritis (CIA) Murine Model

All murine experimental procedures for the CIA model were approved by the University of Manitoba Animal Care Ethics Board, protocol number 11-041. Highly susceptible male DBA/1J mice (∼6 weeks old) were obtained from Jackson laboratories and housed for 2 weeks in the institutional central animal care facility for acclimatization. Experimental mice were divided into four groups, namely, naïve (*n* = 4), saline control (*n* = 4), CIA mice (*n* = 5), and CIA mice treated with buprenorphine (CIA + Bup; *n* = 6). Each group was housed in a separate cage. Mice in the CIA and CIA + Bup groups were challenged with a subcutaneous (s.c.) injection of 100 *μ*g bovine CII emulsified in complete Freund's adjuvant, 1-2 cm from the base of the tail, followed by a boost on day 21 with bovine CII emulsified in incomplete adjuvant, and a LPS challenge (20 *μ*g of LPS per mouse) on day 25, after the first CII challenge [6, 8]. All reagents were purchased from Chondrex Inc. (Redmond, WA, USA). Buprenorphine (30 *μ*g/mL or 0.075 mg/kg body weight) was administered by s.c. injections immediately after CII immunizations (on day 1 and day 21) in the thigh, followed by two more buprenorphine injections every 6 h. Therefore, there were a total of three buprenorphine injections following each CII immunization. All mice were sacrificed on day 29 after the first CII challenge. Disease progression was monitored by measuring joint thickness using digital calipers every 48 h from day 22 onwards, and the disease severity was assessed using a standardized clinical score, as previously described [6, 10] in a blinded manner. Briefly, clinical scores to assess disease severity were as follows: 0 = normal joint, 1 = paw swelling only, 2 = one joint of one limb along with paw swelling, 3 = multiple joints on a limb involved, and 4 = all joints involved or limb fusion. A combined score ranging from 0–16 was assigned to each mouse by summing the scores of each paw as previously described [6, 10].

### 2.2. RNA Isolation and mRNA Expression by Quantitative Real-Time PCR (qRT-PCR)

Mice joints with the bone but devoid of skin and surrounding tissues were collected on the day of sacrifice. These were immediately flash-frozen in liquid nitrogen and stored. The flash-frozen mice joints were homogenized in RNA lysis buffer using a Cole-Parmer LabGEN 125 homogenizer. Total RNA was isolated using an RNeasy kit (Qiagen Inc., ON, Canada) according to the manufacture's protocol. Blood samples from the human cohort were collected in PAXgene tubes and stored at −80°C until used. Total RNA was extracted using a PAXgene™ Blood RNA System Kit according to the manufacturer's protocol. Integrity of total RNA and concentrations were determined using the Nanodrop ND-1000 UV-visible spectrophotometer and Agilent 2100 Bioanalyzer (Agilent Technologies). Total RNA with A_260/280_ ≥ 2.0 and RNA integrity number (RIN) of ≥7.0 was used for qRT-PCR analysis. cDNA was synthesized with total RNA input of 1 *μ*g from the murine samples and 300 ng from human samples, using the SuperScript™ III First-Strand Synthesis SuperMix (Life Technologies, ON, Canada). mRNA expression was determined by qRT-PCR using the SuperScript® III Platinum® SYBR® Green One-Step qRT-PCR Kit (Life Technologies) in an Applied Biosystems® 7300 Real-Time PCR System. Relative fold change was calculated in the murine samples using the standard ΔΔCt method [11] after normalization using a housekeeping gene 18s rRNA. Whereas, the ΔCt values obtained after normalization using a housekeeping gene 18s rRNA were used for correlation of mRNA expression with pain scores, in the human cohort. Primers used for qRT-PCR are listed in Supplementary Table I available online at https://doi.org/10.1155/2017/2515408.

### 2.3. Histology

On the day of sacrifice, the ankle joints of mice were collected and fixed in 10% buffered formalin and decalcified using 10% EDTA for 14 days followed by dehydration in increasing concentrations of ethanol. The tissues were embedded in paraffin and serial sagittal sections (5 *μ*m) were obtained. The sections were stained with hematoxylin and eosin (H&E) to monitor cellular infiltration in the joints. Safranin-O stain was used to stain proteoglycans as an indicator of cartilage in the joints. The images were processed with a Zeiss imager M2 using the Zen 2011 software. The stained sections were scored quantitatively in a blinded manner by 3 independent observers. Briefly, histology scores to assess the extent of cellular infiltration and joint integrity were evaluated as follows: 0 = normal synovium, 1 = synovial membrane hypertrophy and cell infiltration, 2 = pannus formation and cartilage erosion, 3 = major erosion of the cartilage, and 4 = loss of joint integrity [12].

### 2.4. Human Early Rheumatoid Arthritis Cohort

Subjects selected for this study had less than one year of clinical synovitis involving at least one joint. The study was approved by the institutional Research Ethics Board. Clinical characteristics of the human cohort are summarized in Table 1. RA diagnosis was determined using the 2010 ACR/EULAR criteria for RA. Baseline arthritis activity (Disease Activity Score DAS28-CRP-3 variable) and function (modified Heath Assessment Questionnaire (mHAQ) were recorded. Pain scores were evaluated for each patient using a visual analogue scale (VAS) 0–10 as previously described [13]. A rheumatoid factor was measured by nephelometry in the hospital laboratory; anticyclical citrullinated peptide (CCP) was measured by ELISA (INOVA) and positivity recorded as per the manufacturer's recommendations. Baseline DMARD-naïve serum from 168 subjects was used to determine the correlation of serum MMP-3 and IFN-*γ* with pain. Baseline blood samples were collected in PAXgene tubes from 18 DMARD-naïve early RA subjects and were used to isolate RNA and determine gene expression by qRT-PCR.

### 2.5. Evaluation of Serum Cytokines, Anticollagen Antibodies, and Human MMP-3

Blood was collected through cardiac puncture from the murine model on the day of sacrifice. The serum obtained was aliquoted and stored at −20°C until used. The serum circulatory levels of murine cytokines (IFN-*γ*, TNF-*α*, IL-1*β*, IL-2, IL-4, IL-5, IL-6, IL-17A, and IL-10) were analyzed using the V-PLEX proinflammatory panel 1 (mouse) by a multiplex Meso Scale Discovery (MSD) platform (Meso Scale Discovery, Rockville, MD, USA) employing the manufacturer's protocol. The data was analyzed using Discovery Workbench 4.0 software (Meso Scale Discovery). The serum circulatory levels of mouse anticollagen antibodies and bovine anticollagen antibodies were determined by ELISA using the mouse anti-mouse type II collagen IgG antibody assay kit and the anti-bovine type II collagen IgG antibody assay kit (Chondrex Inc. Redmond, WA, USA) according to the manufacturer's protocol. Serum levels of human MMP-3 were monitored by ELISA and IFN-*γ* was evaluated using the luminex platform. Serial dilutions of the recombinant cytokines were used to establish a standard curve to determine cytokine concentrations.

### 2.6. Statistical Analysis

GraphPad Prism 5 software was used to analyze the data. Statistical significance was determined by Kruskal-Wallis one-way analysis of variance (ANOVA) followed by Dunn's post hoc test and by Mann–Whitney *U* test to determine the *p* values between the groups in the CIA model. Correlation analyses between pain scores and expression of the selected molecular markers in the human cohort were performed by Spearman's correlation analysis. Linear regression assessed associations of human pain VAS scores and molecular markers while controlling for disease activity using either the DAS28CRP3var or the patient-reported global assessment of disease activity. A *p* value of <0.05 was considered to be statistically significant.

## 3. Results

### 3.1. Buprenorphine Did Not Alter CIA-Induced Morphological Changes of the Joint

**Figure 1:**
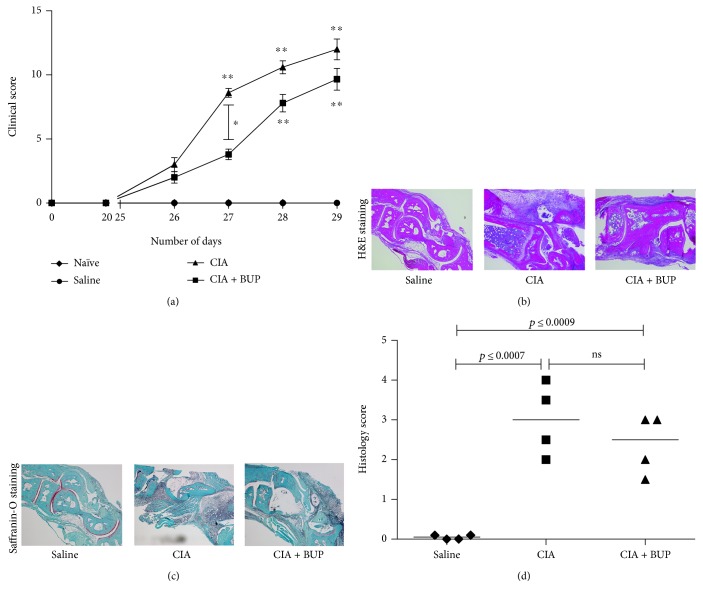
Effect of buprenorphine administration on CIA-induced morphological changes of the joint. DBA/1 mice were challenged with bovine CII, and buprenorphine was administered immediately after CII immunizations followed by two additional buprenorphine injections every 6 h. (a) Clinical scores monitored daily from day 25 to day 29 after the first CII challenge. Mice were sacrificed on day 29 after the first CII challenge. Paraffin-embedded sagittal sections (5 *μ*m) of hind ankle joints collected on day 29 were stained with (b) H&E to assess cellular infiltration and (c) safranin-O to detect cartilage proteoglycan. Images shown are representative of sections from naïve (*n* = 4), CIA (*n* = 5), and CIA + Bup (*n* = 6) mice. Images were processed using a Zeiss imager M2 using the Zen 2011 software. (d) The histology score was determined using a standardized scoring scale as described in the methods. Kruskal-Wallis one-way analysis of variance (ANOVA) followed by Dunn's post hoc test was used to determine the significance, and Mann–Whitney *U* test was used to determine the *p* values between the groups (^∗^*p* < 0.05, ^∗∗^*p* < 0.01); *p* < 0.05 was considered to be statistically significant; ns: not significant.

The murine model of CIA exhibits phenotypic features similar to human RA such as swelling and inflammation at the affected joints [7]. In this study, swelling of the paw, joints, and digits was measured in the CIA murine model using a digital caliper, and disease severity was assessed with a standardized clinical score as previously described by us [6]. Clinical scores were monitored daily from day 22 after the first CII challenge to assess the development of disease severity. Both CIA mice and CIA + Bup mice showed significant swelling and higher clinical scores from day 27 onward (48 hr after LPS challenge) compared to the naïve and saline control mice (Figure 1(a)). Administration of buprenorphine delayed the development of disease severity as indicated by significant difference in clinical scores between the CIA and CIA + Bup groups on day 27 after the first CII challenge (Figure 1(a)). However, there was no significant difference in clinical scores between the CIA and CIA + Bup groups on days 28 and 29 after the first CII challenge (Figure 1(a)). Therefore, we performed all other assessments on day 29 (day of sacrifice) to evaluate if there were any differences in the expression of molecular markers and histopathology between the CIA and CIA + Bup mice, despite no significant difference in the clinical symptoms between these two groups. Histological assessment showed significant cellular infiltration, tissue damage, and loss of joint integrity, in both CIA and CIA + Bup mice (Figure 1(b)). Safranin-O staining for cartilage proteoglycan demonstrated proteoglycan degradation in both CIA and CIA + Bup mice (Figure 1(c)). The staining sections showed that cellular infiltration and cartilage degradation may be less severe in the CIA + Bup mice compared to the CIA mice, which was also indicated by the histology scores, with a median score of 4 for the CIA mice and median score of 3 for the CIA + Bup mice (Figure 1(d)). However, there was no statistically significant difference between the clinical and histology scores between the CIA and the CIA + Bup mice on the day of sacrifice (Figure 1()). These results suggest that administration of buprenorphine did not significantly change the overall morphological assessments in the murine model of CIA, on the day of sacrifice when the samples were collected for molecular evaluation.

### 3.2. Buprenorphine Administration Altered Synovial Gene Expression in the CIA Mice

**Figure 2:**
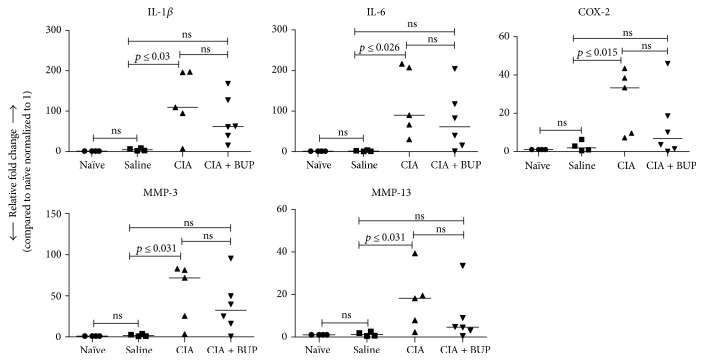
Buprenorphine administration altered gene expression of inflammatory markers in the synovial joint of the CIA mice. Mice were sacrificed on day 29 after the first CII challenge in the CIA model, and ankle joints were collected. The mRNA expression of *cox-2*, *mmp-3*, *IL-1β*, and *IL-6* were evaluated by qRT-PCR. Relative fold changes were calculated compared to the expression in the naïve mice normalized to 1, using the standard ΔΔCt method, after normalization with 18s RNA expression. Kruskal-Wallis one-way analysis of variance (ANOVA) followed by Dunn's post hoc test was used to determine the significance, and Mann–Whitney *U* test was used to determine the *p* values between the groups; *p* < 0.05 was considered to be statistically significant; ns: not significant.

**Figure 3:**
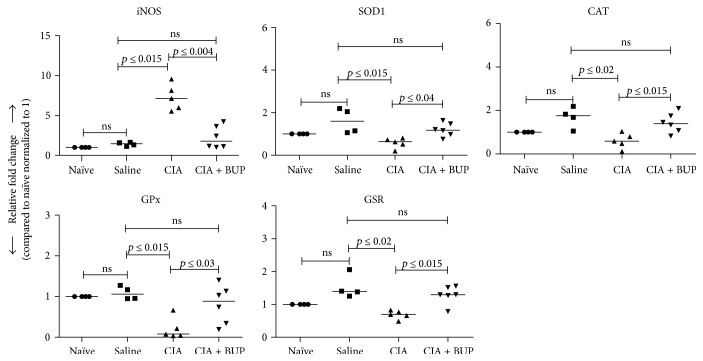
Buprenorphine administration altered gene expression of oxidative stress markers in the synovial joint of the CIA mice. Mice were sacrificed on day 29 after the first CII challenge in the CIA model, and ankle joints were collected. The mRNA expression of an oxidative stress marker *iNOS* and antioxidant markers *sod1*, *cat*, *gpx*, and *gsr* were evaluated by qRT-PCR. Relative fold changes were calculated compared to the expression in the naïve mice normalized to 1, using the standard ΔΔCt method, after normalization with 18s RNA expression. Kruskal-Wallis one-way analysis of variance (ANOVA) followed by Dunn's post hoc test was used to determine the significance, and Mann–Whitney *U* test was used to determine the *p* values between the groups; *p* < 0.05 was considered to be statistically significant; ns: not significant.

Molecular indicators of inflammation and oxidative stress are used as readouts in animal models of inflammatory arthritis to delineate underlying mechanisms of disease progression and response to candidate therapy [14]. In this study, we demonstrated that there was a significant increase in the mRNA expression of proinflammatory mediators such as *cox-2* (~10-fold), *mmp-3* (~25-fold), *IL-1β* (~16-fold), and *IL-6* (>75-fold), in the synovial tissues of the CIA mice compared to the saline control mice (Figure 2). Whereas, an increase in the mRNA expression of these molecular markers was not statistically significant in the CIA + Bup mice compared to the saline control mice (Figure 2). In addition, the mRNA expression of inducible nitric oxide synthase (*iNOS*), a critical oxidative stress marker, was significantly increased in the CIA mice, but was not increased in the CIA + Bup mice, compared to the saline control mice (Figure 3). Furthermore, the expression of *iNOS* was significantly decreased in the CIA + Bup mice compared to the CIA group (Figure 3). As iNOS accounts for increased nitric oxide, a free radical contributing to oxidative damage, we also evaluated the expression of specific antioxidant enzymes. We showed that the mRNA expressions of key antioxidant enzymes, namely, superoxide dismutase (*sod1*), catalase (*cat*), glutathione peroxidase (*gpx*), and glutathione reductase (*grs*), were decreased in the CIA mice compared to the saline control mice, but not in the CIA + Bup mice (Figure 3). Moreover, the mRNA expressions of *cat* and *grs* were significantly higher (*p* < 0.05), while *sod1* and *gpx* showed a similar trend toward higher expression (*p* < 0.08), in the CIA + Bup mice compared to the CIA mice (Figure 3). Taken together (Figures 2 and 3), these results indicate that administration of buprenorphine interferes with the process of both oxidative stress and inflammation in the murine model of CIA.

### 3.3. Buprenorphine Administration Altered Serum Cytokine Production in the CIA Mice

**Figure 4:**
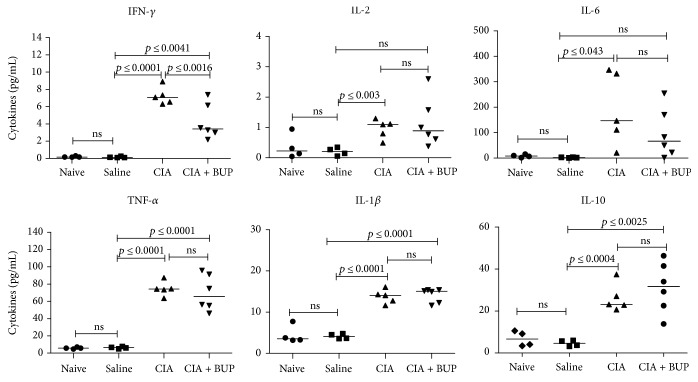
Buprenorphine administration altered serum cytokine production in the CIA mice. Serum was collected from the CIA mice with and without buprenorphine administration on day 29 after the first CII challenge (day of sacrifice). Circulating levels of a panel of cytokines (IFN-*γ*, IL-2, IL-6, TNF-*α*, IL-1*β*, and IL-10) were analyzed using the V-PLEX proinflammatory panel 1 (mouse) by multiplex Meso Scale Discovery (MSD) platform (Meso Scale Discovery, Rockville, MD, USA) employing the manufacturer's protocol. The data was analyzed using Discovery Workbench 4.0 software (Meso Scale Discovery). Kruskal-Wallis one-way analysis of variance (ANOVA) followed by Dunn's post hoc test was used to determine the significance, and Mann–Whitney *U* test was used to determine the *p* values between the groups; *p* < 0.05 was considered to be statistically significant; ns: not significant.

We further monitored the serum concentrations of a panel of cytokines (IFN-*γ*, TNF-*α*, IL-1*β*, IL-2, IL-4, IL-5, IL-6, IL-17A, and IL-10) in the murine model, to enumerate if administration of buprenorphine also altered cytokine production in the CIA mice. The CIA mice had significantly elevated serum concentrations of cytokines IFN-*γ*, IL-2, IL-6, TNF-*α*, IL-1*β*, and IL-10 compared to the saline control mice (Figure 4). In contrast, the serum concentrations of IL-2 and IL-6 were not significantly increased in the CIA + Bup mice compared to the saline control mice (Figure 4). Moreover, serum concentration of the proinflammatory cytokine IFN-*γ* was significantly lower in the CIA + Bup mice compared to the CIA mice (Figure 4). Even though the serum concentration of the anti-inflammatory cytokine IL-10 was higher in the CIA + Bup mice compared to the CIA mice, the difference between the two groups was not statistically significant. These results indicate that administration of buprenorphine also alters the production of certain serum cytokines in the CIA murine model.

### 3.4. Buprenorphine Administration Did Not Alter Anticollagen Type II (CII) Antibodies in the CIA Mice

CII is exclusively expressed in the joints and considered an autoantigen of RA [12, 15]. Immunization of DBA/1J mice with heterologous bovine CII results in the production of antibodies against the bovine CII (immunizing antigen) and the murine CII autoantigen, in the murine model of CIA [6, 14]. Therefore, in this study, we monitored the circulating serum concentrations of antibodies both to bovine CII and to murine CII by ELISA. Both CIA mice and CIA + Bup mice had significantly elevated levels of anti-bovine CII and anti-mouse CII antibodies compared to the saline control mice (Supplementary Figure 1). There was no statistically significant difference in the serum levels of these antibodies in the CIA mice compared to the CIA + Bup mice (Supplementary Figure 1).

### 3.5. Serum Levels of MMP-3 and Gene Expressions of Antioxidants Significantly Correlated with Pain Scores in a Human Cohort of Early RA

**Figure 5:**
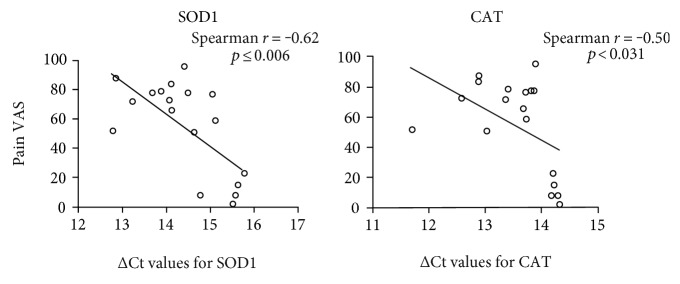
Correlation analyses of gene expression of oxidative stress markers with pain scores in the human cohort. Baseline blood samples were collected in PAXgene tubes from 18 DMARD-naïve early RA subjects and were used to isolate RNA and determine gene expression by qRT-PCR. Each circle in the graph represents the ∆Ct values of individual samples after normalization with 18s RNA expression. Spearman correlation analysis was used to determine the correlation between pain VAS and specific gene expression. A *p* value of <0.05 was considered to be statistically significant.

As buprenorphine is an analgesic known to alleviate pain, we examined the correlation of systemic expression of molecular indicators identified to be altered by buprenorphine in the CIA mice with pain scores (VAS) in a human cohort of early RA. We specifically examined serum concentrations of IFN-*γ* and MMP-3, and whole blood mRNA expression of *sod1*, *cat*, *gpx*, *grs*, and *iNOS* (Figures 2(), 3(), and 4()), with pain scores in a human cohort of early RA. Circulating serum levels of MMP-3 showed a significant correlation with pain scores (30%) and mHAQ scores (40%), in the human cohort (Table 2). However, the associations between MMP-3 and either pain or mHAQ were no longer significant after adjusting for disease activity. mRNA expression of oxidative markers *gpx* and *grs* did not show a significant linear correlation with pain scores, and mRNA expression of *iNOS* was not detected by qRT-PCR in the whole blood samples obtained from the human cohort of early inflammatory arthritis, whereas blood mRNA expression of antioxidant enzymes *sod1* and *cat* showed significant linear correlation of >60% (*p* < 0.006) and 50% (*p* < 0.03) respectively, with pain scores in the human cohort (Figure 5() and Table 2). Moreover, the association of patient reported pain and antioxidant *sod1* expression remained significant even after adjusting for RA disease activity using the composite disease activity score (Supplementary Table II).

## 4. Discussion

Analgesics such as buprenorphine are commonly administered in experimental animals during disease progression to manage pain. Previous studies have demonstrated some effects of buprenorphine on models of inflammatory arthritis. For example, Hall et al. showed that buprenorphine inhibits osteoclastic bone resorption in an adjuvant-induced arthritis rat model [16]. Oral administration of buprenorphine was also shown to modulate the disease pathogenesis of streptococcal cell wall polymer-induced arthritis in Lew/SSN rat [9]. Bas et al. demonstrated the alleviation of mechanical hypersensitivity in buprenorphine-administered QB mice during the inflammatory and postinflammatory phases of arthritis [7]. Despite these previous studies which suggest that buprenorphine may intervene in the disease process of arthritis, buprenorphine is recommended to be administered as an analgesic in animal models to manage pain. Therefore, in this study, we interrogated the effect of buprenorphine administration on disease-associated molecular indicators of inflammation and oxidative stress in a murine model of CIA, which is a commonly used preclinical model of RA.

The expression and activation of proinflammatory mediators, both at the synovial joints and in circulation, are known to intensify the disease severity in RA [17, 18]. Increased production of proinflammatory mediators further induces the expression of oxidative stress markers such as COX-2 and iNOS, which in turn influences the production of prostaglandins and nitric oxide, together promoting inflammation and oxidative damage of the arthritic joint [7]. Furthermore, expressions of key endogenous antioxidant enzymes such as SOD, CAT, Gpx, and GSR are also induced, which aims to neutralize reactive oxygen/nitrogen species, thus functioning as an endogenous defense mechanism against oxidative stress [19]. In this study, we showed that administration of buprenorphine altered the expression of inflammatory cytokines, metalloproteinases, oxidative stress markers, and antioxidant enzymes, in a murine model of CIA. Administration of buprenorphine intervened in the significant increase in synovial gene expression of inflammatory mediators such as cytokines *IL-6* and *IL-1β*, *cox-2*, and metalloproteinases *mmp-3* and *mmp-13*, in the CIA mice. Furthermore, the serum concentration of IFN-*γ* was significantly decreased by administration of buprenorphine in the CIA mice. Administration of buprenorphine had a significant effect on oxidative stress-related molecular markers in the CIA mice; synovial gene expression of an oxidative stress marker *iNOS* was significantly lowered, and that of antioxidant enzymes *cat* and *grs* were significantly increased, by buprenorphine administration in the CIA mice. Our results demonstrated that administration of buprenorphine as an analgesic interfered in the expression of molecular indicators of inflammation and oxidative stress in a preclinical murine model of RA.

Although molecular markers of inflammation and oxidative stress were significantly altered by administration of buprenorphine in the CIA mice, morphological characteristics of arthritis as indicated by clinical and histology scores did not show significant difference on the day of sacrifice and evaluation of molecular markers. However, it should be noted that administration of buprenorphine delayed the development of disease severity as indicated by the lower trend of clinical scores prior to the day of sacrifice. Furthermore, in this study, we used a low dose of buprenorphine (0.075 mg/kg body weight) compared to that in the previous studies demonstrating buprenorphine interference in the pathology of disease development. For example, mitigation of arthritis-associated mechanical hypersensitivity was demonstrated in a study that administered buprenorphine at a dose of 0.1 mg/kg body weight [7]. Similarly, buprenorphine administrated at a high dose of 2 mg/kg in Lew/SSN rats showed anti-inflammatory effects and pathophysiological changes in a streptococcal cell wall polymer-induced arthritis [9]. Our findings clearly demonstrate that buprenorphine even at lower doses, and at a time point when there are no observed clinical symptoms, interferes in the expression of inflammatory and oxidative stress molecular markers in a murine model of CIA. Therefore, analgesics such as buprenorphine used in murine models of inflammatory arthritis may be a confounding factor, as it may result in additive or synergistic effects on the expression of molecular markers of inflammation and/or oxidative stress with potential candidate therapeutics under evaluation. This caveat needs to be considered while using buprenorphine as an analgesic in animal models of inflammatory arthritis, especially in studies aimed at defining the molecular mechanisms underlying activity of new candidate therapies.

Inflammation, oxidative stress, and pain are inherent characteristics that reflect the disease severity in inflammatory arthritis. A recent study has shown that treatments used to reduce pain in RA, such as laser acupuncture, alleviate oxidative stress and inflammation in patients [20]. Also, a previous study has demonstrated that pain-reducing anesthesia impedes oxidative stress in human term placenta [21]. In this study, we have shown that the analgesic buprenorphine used to control pain significantly altered the expression of inflammatory and oxidative stress-related molecular markers in the murine model of CIA. Therefore, we speculated that the molecular markers altered by buprenorphine administration in the CIA mice may serve as objective readouts for the evaluation of pain. To address this, we performed correlation analyses of the expression of the selected biomarkers with pain scores in a human cohort of early arthritis. We showed that the serum concentrations of MMP-3 had a 30% linear correlation (*p* < 0.001) with pain scores in the human cohort. This is consistent with the previous study that has demonstrated that the concentration of MMP-3 in synovial fluid directly correlates with the visual analogue score for pain in patients undergoing knee arthroscopy [22]. MMPs are also shown to regulate the development of neuropathic pain in the early and late phase [23]. As it is challenging to accurately measure either the activity or the levels of oxidative enzymes from serum that has been stored in the freezer, we evaluated the mRNA expression of the oxidative stress-related markers in the human cohort. We demonstrated that the mRNA expression of antioxidant enzymes *sod1* and *cat* from whole blood had a significant linear correlation of more than 50% with pain scores, in a human cohort of arthritis. The expression of these antioxidant enzymes negatively correlated with pain scores in the human cohort. Our results are consistent with the previous studies that have demonstrated that the levels of SOD1 and CAT enzymes are altered in chronic neck pain and in the human term placenta [21, 24]. Moreover, the mRNA expression of *sod1* was significantly associated with pain scores even after correcting for disease activity, thus providing a potential objective marker for pain that is independent of disease activity in early RA. Our results suggest that the biomarkers defined in this study may be useful as objective readouts for pain, and this opens up a new avenue for future research to define a biosignature for pain.

In summary, results from this study suggest that buprenorphine alters the expression of molecular markers associated with the processes of inflammation and oxidative stress in a preclinical murine model of RA. The biomarkers altered by the exogenous administration of buprenorphine in the murine model of CIA included inflammatory cytokines such as IFN-*γ*, metalloproteinases (e.g., MMP-3), markers of oxidative stress (iNOS), and antioxidant enzymes (e.g., SOD1 and CAT). Therefore, it is important to consider the interference of buprenorphine in studies evaluating the anti-inflammatory or antioxidant properties of candidate therapeutics in animal models of inflammatory arthritis.

## Supplementary Material

Supplementary Table I: Summary of primers used for quantitative real-time PCR. Supplemental Table II: Linear regression models predicting pain VAS. Supplementary Figure 1: Anti-collagen type II (CII) antibodies in the CIA and CIA+ BUP mice indicate collagen driven disease development. DBA/1 mice were challenged with bovine CII i.d, and buprenorphine was administered by s.c. injections immediately after CII immunizations in the thigh, followed by two more buprenorphine injections every 6 h. Mice were sacrificed on day 29 after the first collagen challenge, and blood was collected to isolate serum. The concentrations of (A) anti-mouse collagen type II antibodies and (B) anti-bovine collagen type II antibodies were monitored in the serum by ELISA. Kruskal-Wallis one way analysis of variance (ANOVA) followed by Dunn's posthoc test was used to determine the significance, and Mann-Whitney U test was used to determine the p-values between the groups. A p-value of <0.05 was considered to be statistically significant.

## Figures and Tables

**Table 1:** Clinical characteristics of the human early rheumatoid arthritis cohort.

	*n* = 168
Age at baseline (mean; SD)	47 (14)
Gender (% female)	77%
Met RA criteria^∗^ (%)	66%
RF (% positive at >40 iu)	52%
CCP (% positive)	46%
Seropositive (RF or CCP)	67%
DAS28 CRP-3 variable (median)	4.1 (3.2, 4.8)
mHAQ (median)	0.5 (0.13, 1.0)
Pain VAS (median)	47 (24, 69)

^∗^2010 ACR/EULAR criteria for RA.

**Table 2:** Correlation analyses of expression of molecular markers with pain scores in the human cohort.

	Pain VAS	DAS28	HAQ	IFN-*γ*	MMP-3	SOD1	CAT
Pain VAS	—	0.48	0.58	−0.09	0.3	−0.62	−0.50
		*p* < 0.0001	*p* < 0.0001	NS	*p* < 0.0001	*p* ≤ 0.006	*p* ≤ 0.0317
		*n* = 165	*n* = 163	*n* = 135	*n* = 168	*n* = 18	*n* = 18

DAS28		—	0.54	−0.001	0.04	−0.09	−0.35
			*p* < 0.0001	NS	*p* < 0.0001	NS	NS
			*n* = 161	*n* = 133	*n* = 168	*n* = 16	*n* = 16

HAQ			—	−0.1	0.4	−0.32	−0.53
				NS	*p* < 0.0001	NS	*p* = 0.04
					*n* = 168	*n* = 15	*n* = 15
