# Supplementary material for: Buprenorphine Alters Inflammatory and Oxidative Stress Molecular Markers in Arthritis
Source: Mediators Inflamm. 2017 May 9;2017:2515408. doi: 10.1155/2017/2515408 (PMC5441125; doi:10.1155/2017/2515408)

Supplementary Information

Supplementary Table I: Summary of primers used for quantitative real-time PCR

Gene	Forward primer (5'-3')	Reverse Primer (5'-3')
**Mouse**		
IL-1β	TGTAATGAAAGACGGCACACC	TCTTCTTTGGGTATTGCTTGG
IL-6	GCTACCAAACTGGATATAATCAGGA	CCAGGTAGCTATGGTACTCCAGAA
COX-2	CCAGTCAATCCCTGTTGTTACT	GATCTCAGGGATGGTACATTG
MMP-3	CCAAGTCTAACTCTCTGGAACCTG	AGAGATTTGCGCCAAAAGTG
MMP-13	TGGACCTTCTGGTCTTCTGG	GGCATCCCCACCATAGTTT
iNOS	GGAGCCTTTAGACCTCAACAGA	AAGGTGAGCTGAACGAGGAG
SOD1	CAGGACCTCATTTTAATCCTCAC	TGCCCAGGTCTCCAACAT
CAT	CCTTCAAGTTGGTTAATGCAGA	CAAGTTTTTGATGCCCTGGT
GPx	CAAGTTTTTGATGCCCTGGT	TCGGACGTACTTGAGGGAAT
GSR	CTATGACAACATCCCTACTGTGGT	CCCATACTTATGAACAGCTTCGT
18s rRNA	GCAATTATTCCCCATGAACG	GGGACTTAATCAACGCAAGC
**Human**		
SOD1	TCATCAATTTCGAGCAGAAGG	GCAGGCCTTCAGTCAGTCC
CAT	TCATCAGGGATCCCATATTGTT	CCTTCAGATGTGTCTGAGGATTT
GPx	GGGGACAAGAGAAGTCGAAGA	GCCAGCATACTGCTTGAAGG

GSR	ATGATCAGCACCAACTGCAC	CCCTTGTCATCGGTTTGAAT
iNOS	CTTACGAGGCGAAGAAGGAC	TCAGAGCGCTGACATCTCC
18s rRNA	GTAACCCGTTGAACCCCATT	CCATCCAATCGGTAGTAGCG

Supplemental Table II: Linear regression models predicting pain VAS

Variable	Beta	CI B	P value
Model 1			
SOD1	-20.3	-5.8 - -4.7	0.02
DAS28CRP3var	7.1	-5.5 – 19.8	NS
Model 2			
SOD1	-11.2	-20.2 - -2.2	0.02
Patient global	0.7	0.4 - 0.9	≤0.001
Model3			
CAT	-19.7	-42.2 - 2.9	0.08
DAS28CRP3var	7.4	-7.1 – 21.9	NS
Model 4			
CAT	-11	-27.1 – 0.003	0.05
Patient global	0.72	0.5 - 0.9	≤0.001

Supplementary Figure 1: ***Anti-collagen type II (CII) antibodies in the CIA and CIA+BUP mice indicate collagen driven disease development*.** DBA/1 mice were challenged with bovine CII i.d, and buprenorphine was administered by s.c. injections immediately after CII immunizations in the thigh, followed by two more buprenorphine injections every 6 h. Mice were sacrificed on day 29 after the first collagen challenge, and blood was collected to isolate serum. The concentrations of **(A)** anti-mouse collagen type II antibodies and **(B)** anti-bovine collagen type II antibodies were monitored in the serum by ELISA. Kruskal-Wallis one way analysis of variance (ANOVA) followed by Dunn's *posthoc* test was used to determine the significance, and Mann-Whitney U test was used to determine the p-values between the groups. A p-value of <0.05 was considered to be statistically significant.

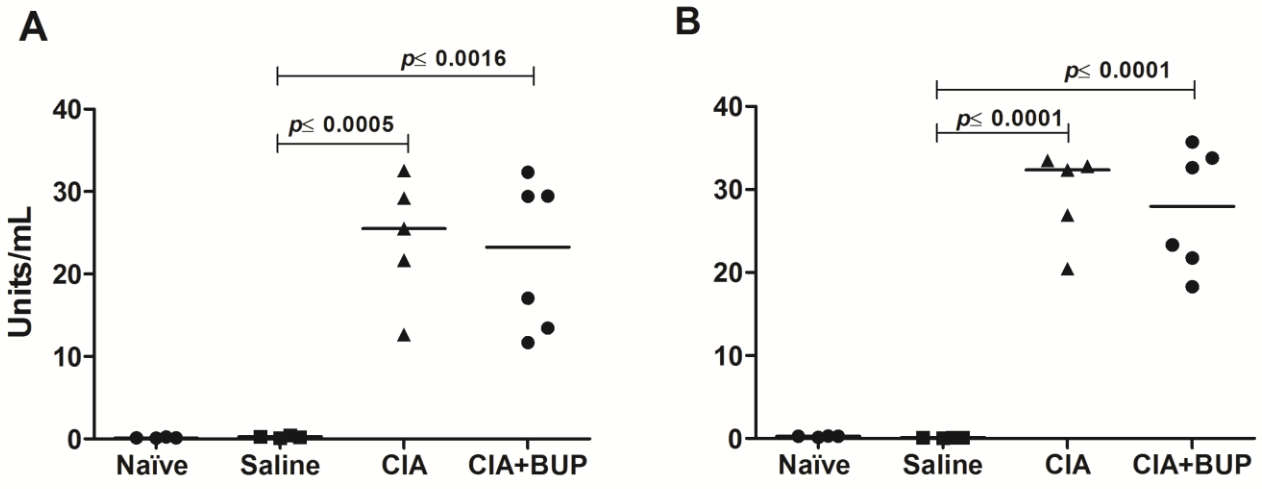

Supplement: Supplementary file 1 — Supplementary Table I: Summary of primers used for quantitative real-time PCR. Supplemental Table II: Linear regression models predicting pain VAS. Supplementary Figure 1: Anti-collagen type II (CII) antibodies in the CIA and CIA+ BUP mice indicate collagen driven disease development. DBA/1 mice were challenged with bovine CII i.d, and buprenorphine was administered by s.c. injections immediately after CII immunizations in the thigh, followed by two more buprenorphine injections every 6 h. Mice were sacrificed on day 29 after the first collagen challenge, and blood was collected to isolate serum. The concentrations of (A) anti-mouse collagen type II antibodies and (B) anti-bovine collagen type II antibodies were monitored in the serum by ELISA. Kruskal-Wallis one way analysis of variance (ANOVA) followed by Dunn's posthoc test was used to determine the significance, and Mann-Whitney U test was used to determine the p-values between the groups. A p-value of <0.05 was considered to be statistically significant. [file 2515408.f1.pdf]
