# Supplementary material for: Buprenorphine Alters Inflammatory and Oxidative Stress Molecular Markers in Arthritis
Source: Mediators Inflamm. 2017 May 9;2017:2515408. doi: 10.1155/2017/2515408 (PMC5441125; doi:10.1155/2017/2515408)
Supplement: Supplementary file 2 [file 2515408.f2.docx]

**Supplementary Information**

**Supplementary Table I: Summary of primers used for quantitative real-time PCR**

| **Gene** | **Forward primer (5’-3’)** | **Reverse Primer (5’-3’)** |
| --- | --- | --- |
| **Mouse** | | |
| IL-1β | TGTAATGAAAGACGGCACACC | TCTTCTTTGGGTATTGCTTGG |
| IL-6 | GCTACCAAACTGGATATAATCAGGA | CCAGGTAGCTATGGTACTCCAGAA |
| COX-2 | CCAGTCAATCCCTGTTGTTACT | GATCTCAGGGATGGTACATTG |
| MMP-3 | CCAAGTCTAACTCTCTGGAACCTG | AGAGATTTGCGCCAAAAGTG |
| MMP-13 | TGGACCTTCTGGTCTTCTGG | GGCATCCCCACCATAGTTT |
| iNOS | GGAGCCTTTAGACCTCAACAGA | AAGGTGAGCTGAACGAGGAG |
| SOD1 | CAGGACCTCATTTTAATCCTCAC | TGCCCAGGTCTCCAACAT |
| CAT | CCTTCAAGTTGGTTAATGCAGA | CAAGTTTTTGATGCCCTGGT |
| GPx | CAAGTTTTTGATGCCCTGGT | TCGGACGTACTTGAGGGAAT |
| GSR | CTATGACAACATCCCTACTGTGGT | CCCATACTTATGAACAGCTTCGT |
| 18s rRNA | GCAATTATTCCCCATGAACG | GGGACTTAATCAACGCAAGC |
| **Human** | | |
| SOD1 | TCATCAATTTCGAGCAGAAGG | GCAGGCCTTCAGTCAGTCC |
| CAT | TCATCAGGGATCCCATATTGTT | CCTTCAGATGTGTCTGAGGATTT |
| GPx | GGGGACAAGAGAAGTCGAAGA | GCCAGCATACTGCTTGAAGG |
| GSR | ATGATCAGCACCAACTGCAC | CCCTTGTCATCGGTTTGAAT |
| iNOS | CTTACGAGGCGAAGAAGGAC | TCAGAGCGCTGACATCTCC |
| 18s rRNA | GTAACCCGTTGAACCCCATT | CCATCCAATCGGTAGTAGCG |

**Supplemental Table II: Linear regression models predicting pain VAS**

| **Variable** | **Beta** | **CI B** | **P value** |
| --- | --- | --- | --- |
| Model 1  SOD1  DAS28CRP3var | -20.3  7.1 | -5.8 - -4.7  -5.5 – 19.8 | 0.02  NS |
| Model 2  SOD1  Patient global | -11.2  0.7 | -20.2 - -2.2  0.4 - 0.9 | 0.02  ≤0.001 |
| Model3  CAT  DAS28CRP3var | -19.7  7.4 | -42.2 - 2.9  -7.1 – 21.9 | 0.08  NS |
| Model 4  CAT  Patient global | -11  0.72 | -27.1 – 0.003  0.5 - 0.9 | 0.05  ≤0.001 |

**
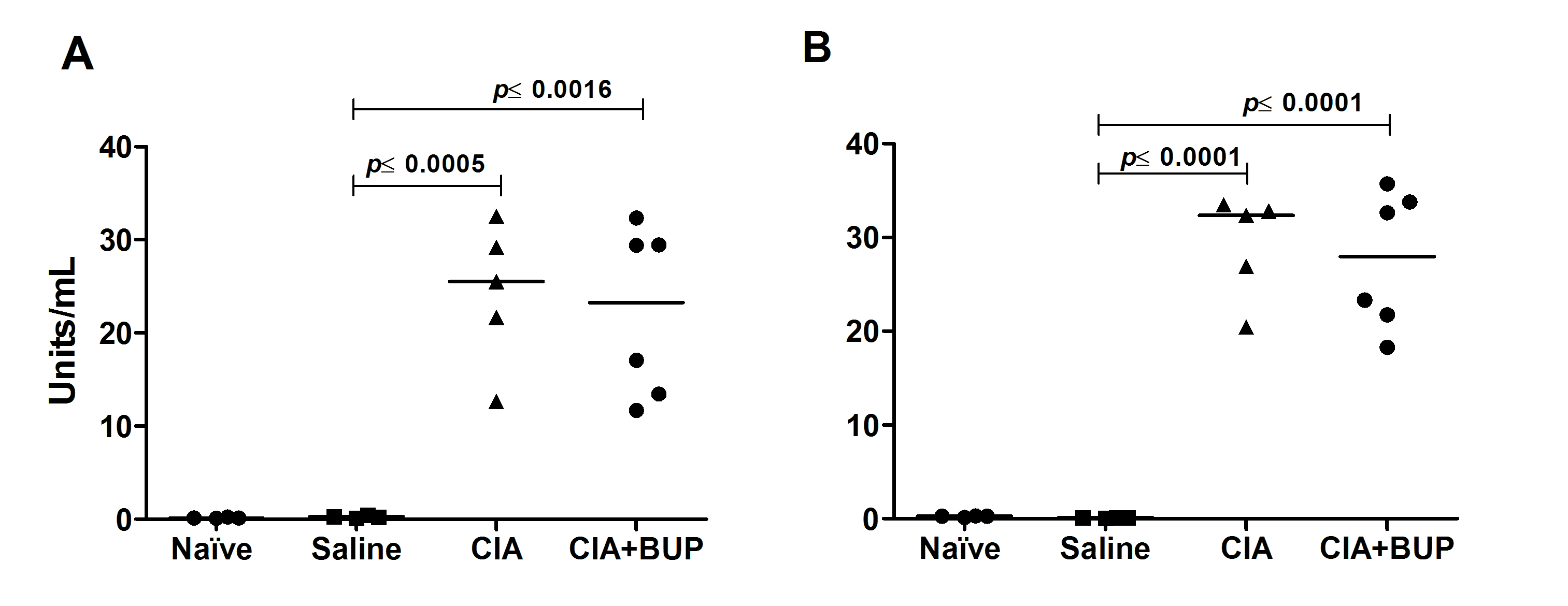
Supplementary Figure 1:** ***Anti-collagen type II (CII) antibodies in the CIA and CIA+BUP mice indicate collagen driven disease development*.** DBA/1 mice were challenged with bovine CII i.d, and buprenorphine was administered by s.c. injections immediately after CII immunizations in the thigh, followed by two more buprenorphine injections every 6 h. Mice were sacrificed on day 29 after the first collagen challenge, and blood was collected to isolate serum. The concentrations of **(A)** anti-mouse collagen type II antibodies and **(B)** anti-bovine collagen type II antibodies were monitored in the serum by ELISA. Kruskal-Wallis one way analysis of variance (ANOVA) followed by Dunn’s *posthoc* test was used to determine the significance, and Mann-Whitney U test was used to determine the p-values between the groups. A p-value of <0.05 was considered to be statistically significant.
